# *Cecropia pachystachya*: A Species with Expressive *In Vivo* Topical Anti-Inflammatory and *In Vitro* Antioxidant Effects

**DOI:** 10.1155/2014/301294

**Published:** 2014-04-30

**Authors:** Natália Ramos Pacheco, Nícolas de Castro Campos Pinto, Josiane Mello da Silva, Renata de Freitas Mendes, Juliana de Carvalho da Costa, Danielle Maria de Oliveira Aragão, Maria Christina Marques Nogueira Castañon, Elita Scio

**Affiliations:** ^1^Laboratory of Bioactive Natural Products, Department of Biochemistry, Federal University of Juiz de Fora, 36036 900 Juiz de Fora, MG, Brazil; ^2^Department of Morphology, Biological Sciences Institute, Federal University of Juiz de Fora, 36036 900 Juiz de Fora, MG, Brazil

## Abstract

*Cecropia pachystachya* is a species traditionally used in Brazil to treat inflammation. This work aims to evaluate the topical anti-inflammatory and antioxidant activities of the methanolic extract of *C. pachystachya* (CPM) and to perform its chemical fingerprint by HPLC-DAD. The topical anti-inflammatory activity was evaluated using the mouse models of acute ear inflammation induced by croton oil, arachidonic acid, capsaicin, EPP, phenol, and chronic inflammation induced by multiple application of croton oil. The *in vitro* antioxidant effect of CPM was investigated using DPPH, reducing power, **β**-carotene bleaching, and TBARS assays. HPLC analysis was performed to quantify the antioxidant phenolics orientin, isoorientin, and chlorogenic acid previously identified in CPM. CPM exhibited significant anti-inflammatory effect in the acute models, in some cases comparable to the reference drugs. Histopathological analysis showed a moderate chronic skin anti-inflammatory effect with decrease in vasodilation, edema, cell infiltration, and epidermal hyperproliferation. It also showed strong *in vitro* antioxidant activity. The contents of orientin, isoorientin, and chlorogenic acid were 66.5 ± 1.8, 118.8 ± 0.7, and 5.4 ± 0.2 µg/mg extract, respectively. The topical anti-inflammatory activity of CPM could be based on its antioxidant properties, although other effects are probably involved, including COX inhibition and other mechanisms.

## 1. Introduction

A high level of reactive oxygen species (ROS) is proposed to contribute to the pathophysiological mechanisms associated with various inflammatory skin disorders [1, 2]. Several anti-inflammatory drugs have recently been shown to have an antioxidant and/or radical scavenging mechanism as part of their activity [3, 4]. In this context, vegetal extracts with antioxidant effects have been established as a therapeutic approach for treating inflammation.

*Cecropia pachystachya* Trécul (Cecropiaceae) is a fast-growing tropical tree species of Central and South America and is abundant throughout Brazil. Popularly known as embaúba, umbaúba, imbaúba, and embaúva, it has been used in folk medicine as diuretic and for the treatment of asthma, cough, hypertension, diabetes, and inflammation [5, 6]. Previous work had shown that* C. pachystachya* produces chlorogenic acid, isoorientin, orientin [7, 8], catechin, epicatechin, isoquercitrin, isovitexin, procyanidin B2 [8], sitosterol, *α*-amirin, and ursolic, pomolic, and oleanolic acids [9].

Recently, the hypoglycemic [7] and oral anti-inflammatory [10] properties of the methanol extract of* C. pachystachya* leaves were demonstrated. Hypotensive [11], cardiotonic, and sedative [12] effects were also described for the leaves aqueous extract. The hexane extract of the leaves exhibited a significant anti-inflammatory effect which was related to sitosterol isolated from that extract [9]. However, no reports on topical anti-inflammatory properties of this species were described.

Hence, the aims of this study were to evaluate the* in vivo* topical anti-inflammatory effects of the methanol extract of* C. pachystachya* leaves in models of acute and chronic skin inflammation in mice, as well as its possible mechanism of action. Also, the* in vitro* antioxidative activity of the extract was evaluated.

## 2. Materials and Methods

### 2.1. Reagents

1,1-diphenyl-2-picrylhydrazyl (DPPH), 2,6-di-*tert*-butyl-4-methylphenol (BHT), *β*-carotene, linoleic acid, Tween 20, quercetin, rutin, ascorbic acid, orientin, isoorientin, chlorogenic acid, croton oil, arachidonic acid, capsaicin, ethyl-phenylpropiolate (EPP), phenol, indomethacin, and dexamethasone were purchased from Sigma (St. Louis, MO, USA). Thiobarbituric acid (TBA) was purchased from Acros Organics (New Jersey, NJ). All solvents used for HPLC analysis were HPLC grade (Tedia Company, Fairfield, USA). Commercial chow was Nuvital, Colombo, PR, BR. All other reagents were of the highest quality available.

### 2.2. Plant Material

The leaves of* Cecropia pachystachya* were collected in Juiz de Fora, Minas Gerais, Brazil, in November, 2010. A voucher specimen (CESJ 46591) was deposited at the Leopoldo Krieger Herbarium of the Federal University of Juiz de Fora.

### 2.3. Preparation of Plant Extract

The dried leaves (60 g) were powdered and macerated with methanol (3 × 200 mL) for five days at room temperature. The extract (CPM) was then concentrated using a rotary evaporator under reduced pressure (yield 26 g) and kept in tightly stoppered bottle under refrigeration.

### 2.4. Chemical Fingerprint and Quantitative Analysis of CPM by HPLC-DAD

Liquid chromatography was performed using an Agilent 1200 machine (Waldbronn, Germany). The sample was applied using an automatic injector and separated on a Zorbax SB-C18 column, 250 mm × 4.6 mm at 25°C. The mobile phase was a gradient of acetonitrile: H_2_O, 7.5 : 92.5, v/v, pH 3.0 with acetic acid for 15 min and acetonitrile : H_2_O, 15 : 85, v/v, pH 3.0 with acetic acid 15–30 min. The flow rate was 1 mL/min and for detection a DAD 330 nm was used. The injection volume for sample and standards was 20 *μ*L. Orientin, isoorientin, and chlorogenic acid were used as standard. To calculate the individual constituents concentration expressed as *μ*g per 1 g dry weight ±SD, the area of individual peaks were integrated and compared to their corresponding standard (each measure in triplicate).

### 2.5. *In Vivo* Anti-Inflammatory Activities of CPM

#### 2.5.1. Animals

Male Swiss mice (25 and 35 g) were housed in a room kept under controlled conditions with temperature maintained at 23°C ± 2°C, on a 12 h light : 12 h dark cycle and free access to water and complete commercial chow. Throughout the experiments, animals were processed according to the ethical guidelines for the care of laboratory animals. The study was approved by the Brazilian College of Animal Experimentation (COBEA—protocol no. 021/2012). Mice were divided in groups of six–eight animals each.

#### 2.5.2. Ear Edema Measurement

Ear thickness was measured using a micrometer (Insize Model 3109–25). To evaluate the ear weight, animals were euthanized, 6 mm diameter ear punch biopsies were collected using a metal punch, and the biopsies were individually weighed on a Shimadzu balance AY220 (Kyoto, Japan). The extent of the edema was expressed as percentage of increase in the ear tissue weight (%), using the following formula: percentage of edema weight (%) = [(*w*_RE_ − *w*_LE_) × 100]/*w*_LE_, where *w*_RE_ is the fragment weight obtained from the right ear (inflamed) and *w*_LE_ is the circle weight obtained from the left ear (noninflamed). The mean of inhibition edema percentage (%) was calculated by comparing to negative control group. CPM was applied topically in 20 *μ*L acetone. Dexamethasone or indomethacin was used as positive controls.

#### 2.5.3. Croton Oil Single Application-Induced Mouse Ear Edema

The croton oil-induced ear edema was determined by the method described by Schiantarelli et al. [13] with minor modifications. Edema was induced on the right ear by topical application of 20 *μ*L of croton oil 5% (v/v) in acetone. After 15 min, CPM (1, 0.5, and 0.1 mg/ear) or dexamethasone (0.1 mg/ear) was applied topically on right ear, while the left ear received 20 *μ*L of vehicle acetone. The thickness of the ears was measured before and 6 h after induction of inflammation. After 24 h, animals were euthanized and 6 mm diameter ear punch biopsies were collected and subjected to histopathological analysis.

#### 2.5.4. Croton Oil Multiple Application-Induced Mouse Ear Edema

This study was conducted for 9 days (days 0–8). Croton oil 5% (v/v) in acetone (20 *μ*L/ear) was applied on the right ear and acetone on the left ear of mice on alternate days. The ear edema was evaluated daily by measuring the ear thickness. On days 4–8, the mice were treated on the inner and outer surfaces of the right ear with CPM (0.5 mg/ear), vehicle acetone (20 *μ*L/ear), or dexamethasone (0.1 mg/ear) twice a day. On day 8, the mice were euthanized and 6 mm diameter ear punch biopsies were collected and weighted [14] and used for histopathological analysis.

#### 2.5.5. Arachidonic Acid-Induced Mouse Ear Edema

Arachidonic acid (AA) ear edema was performed according to Young et al. [15]. AA dissolved in acetone (2 mg/mL) was applied to the inner and outer surfaces of the right ear of mice (20 *μ*L/ear). CPM (1, 0.5, and 0.1 mg/ear) or indomethacin (0.5 mg/ear) was applied topically on right ear, while the left ear received 20 *μ*L of vehicle acetone. The weight of the ears was measured 1 h after induction of inflammation.

#### 2.5.6. Capsaicin-Induced Mouse Ear Edema

Inflammation was induced in mice by applying 20 *μ*L capsaicin 0.02 mg/*μ*L in acetone on the inner and outer surfaces of the right ear. Fifteen minutes before the application of capsaicin, the right ears were topically treated with CPM (0.1 mg/ear) and vehicle acetone. Ear weight was measured 30 min after capsaicin application [16].

#### 2.5.7. Ethyl Phenylpropiolate- (EPP-) Induced Mouse Ear Edema

EPP ear edema was performed according to Giner et al. [17] and Recio et al. [18]. EPP dissolved in acetone (50 mg/mL) was applied to the inner and outer surfaces of the right ear of mice (20 *μ*L/ear). CPM (1, 0.5, and 0.1 mg/ear) or dexamethasone (0.1 mg/ear) was applied topically on right ear, while the left ear received 20 *μ*L of vehicle acetone 16 h before and immediately after EPP application. The weight of the ears was measured 1 h after induction of inflammation.

#### 2.5.8. Phenol-Induced Mouse Ear Edema

Inflammation was induced in mice by applying on the inner and outer surfaces of the right ear 20 *μ*L 10% phenol (v/v) in acetone. CPM (1, 0.5, and 0.1 mg/ear) or dexamethasone (0.1 mg/ear) were applied topically on right ear immediately after the application of the irritant agent. The left ear received 20 *μ*L of vehicle acetone. Ear weight was measured 2 h after phenol application [19].

#### 2.5.9. Histopathology

Ear biopsies from croton oil-induced single and multiple application mouse ear edema were collected and fixed in 70% ethanol for 24 h and then preserved in 10% formalin. Subsequently, the ears were dehydrated, blocked in paraffin, and then sectioned with a microtome (4 *μ*m). The cross-sections were stained with hematoxylin and eosin for the evaluation of leukocyte infiltration, vasodilatation, and edema intensity. Epidermal hyperproliferation was also evaluated in the chronic model. A representative area was selected for qualitative light microscope analysis (100 and 400 x magnification).

### 2.6. *In Vitro* Antioxidant and Free Radical-Scavenging Activity Assays

#### 2.6.1. DPPH Assay

The free radical scavenging activity of CPM solutions in methanol was determined based on their ability to react with stable DPPH free radical [20]. The antioxidant activity of the samples was expressed as IC_50_ (inhibitory concentration), which was defined as the concentration (in *μ*g/mL) of sample required to inhibit the formation of DPPH radicals by 50%. Quercetin and rutin were used as positive control.

#### 2.6.2. Reducing Power

The reducing power was determined by the method of Oyazu [21]. Ascorbic acid was used as reference substance. EC_50_ (effective concentration) values (*μ*g/mL) were calculated and indicated the effective concentration at which the absorbance was 0.5 for reducing power.

#### 2.6.3. *β*-Carotene/Linoleic Acid Bleaching Assay

Antioxidant activity was also determined by using the *β*-carotene bleaching test [22] at concentrations of 38.46 to 1.20 *μ*g/mL of CPM. Quercetin was used as positive control, at the same concentration as samples. All samples were assayed in triplicate and the results were expressed in IC_50_ which is the sample concentration providing 50% inhibition of linoleic acid oxidation.

#### 2.6.4. Thiobarbituric Acid Reactive Substances (TBARS) Assay

The products of oxidation were quantified using thiobarbituric acid reactive substances (TBARS) assay as described by Uchiyama and Mihara [23] with some modifications. Briefly, a mixture of 100 g of ground beef and 67 mL of distilled and deionized water with 7.5 mg of CPM (dissolved in methanol) were thoroughly blended at the high setting at room temperature. Control samples contained only beef, water, and methanol or 7.5 mg BHT (dissolved in methanol) were used. This mixture was blended until a smooth homogenate was formed. The samples were transferred to amber jars and stored in a 5°C cold room over a period of 5 days. After this time, the percentage of beef oxidation was measured and the results were expressed in % oxidation inhibition. A calibration curve was prepared using an MDA standard, reacting with the TBA/phosphoric acid solution, and measuring the absorbance spectrum of 535 nm.

### 2.7. Statistical Analysis

The results are expressed as mean ± standard error of mean (s.e.m.). The comparison between groups was assessed by one-way analysis of variance (ANOVA) followed by Student-Newman-Keuls test or by two-way ANOVA followed by Bonferroni test (repeated measures) when appropriate. Values of *P* < 0.05 were accepted as statistically significant compared to negative control group. The software GraphPad Prism 5.0. was used for statistical analyses. For the* in vitro* antioxidant experiments, the data were expressed as the mean ± standard deviation (SD). The differences between samples and the controls were evaluated by ANOVA followed by test* t*-Student.

## 3. Results 

### 3.1. HPLC Analysis of CPM

**Figure 1:**
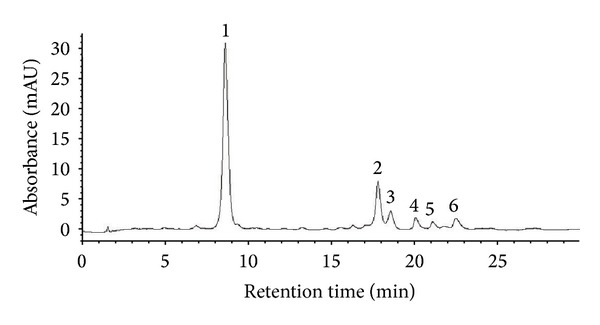
HPLC fingerprint chromatogram of the methanol extract of* C. pachystachya* leaves (CPM). The peaks were indicating as follows: (1) chlorogenic acid; (2) isoorientin; (3) orientin. Peaks 4, 5, and 6 are of nonidentified flavonoids. DAD 330 nm was used.

Orientin, isoorientin, and chlorogenic acid have been identified by their retention time and UV absorbance of purified standards. According to the plot of peak-area ratio (*y*) versus concentration (*x*, *μ*g/g extract), the regression equations of the three constituents and their correlation coefficients (*r* ^2^) were determined as follows: *y* = 1125*x* − 10.58  (*r* ^2^ = 0.982) for orientin, *y* = 793.5*x* + 16.09  (*r* ^2^ = 0.989) for isoorientin, and *y* = 27440*x* + 511.5  (*r* ^2^ = 0.995) for chlorogenic acid. The relative amounts of these compounds in CPM were 66.5 ± 1.8 *μ*g/g for orientin, 118.8 ± 0.7 *μ*g/g for isoorientin, and 5.4 ± 0.2 *μ*g/g for chlorogenic acid. Besides, the UV spectra recorded by DAD pointed out some peaks of nonidentified flavonoids (Figure 1).

### 3.2. *In Vivo* Topical Anti-Inflammatory Activities of CPM

#### 3.2.1. Effect of CPM on Croton Oil Single Application-Induced Ear Edema

**Figure 2:**
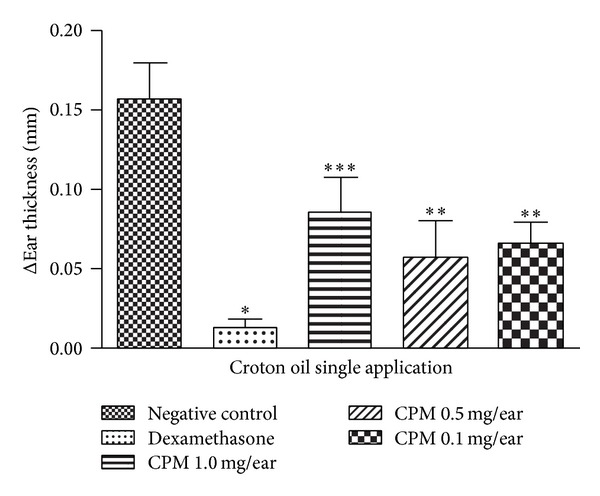
Effect of CPM single application croton oil-induced mouse ear edema. Dexamethasone was used as positive control. Statistical analysis: one-way ANOVA followed by Newman-Keuls test (*n* = 6–8). *P < 0.05, **P < 0.01,  and ***P < 0.001 compared to negative control group.

According to the croton oil single application-induced ear edema, the negative control group demonstrated the greatest degree of edema, while the mice group pretreated with CPM demonstrated significant reduction of the edema in all concentrations tested, being more active at low doses as 0.5 mg/ear and 0.1 mg/ear, with 64% and 58% reduction of edema, respectively. At 1 mg/ear, CPM reduced the edema by 45%. The positive control (dexamethasone 0.1 mg/ear) caused a reduction of the edema by 92% (Figure 2).

#### 3.2.2. Effect of CPM on Croton Oil Multiple Application-Induced Ear Edema

**Figure 3:**
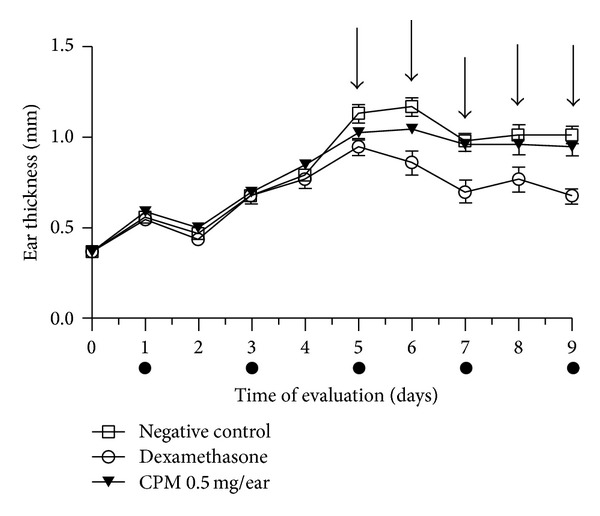
Effect of CPM on croton oil multiple application-induced mouse ear edema showing the time-response curve of effect during 9 days. Dark points under times indicate when the croton oil application occurred. The thickness of the ear was measured daily, using a digital micrometer. At the time 120 h (fifth day of the experiment) the ear of the animals received vehicle acetone (negative control), dexamethasone (positive control), or CPM 0.5 mg/ear, twice a day (arrows indicate the days of treatment which treatment). Statistical analysis: two-way ANOVA followed by Bonferroni test (*n* = 6–8). ***P < 0.001 compared to negative control group.

When analyzing the croton multiple application-induced ear edema (a chronic inflammatory model), the application of CPM 0.5 mg/ear twice a day during 4 days did not reduce the edema when compared to the negative control group 24 h after the beginning of the treatment and on subsequent days, while positive control group showed an edema reduction of 34% (Figure 3).

#### 3.2.3. Effect of CPM on AA-Induced Mice Ear Edema

**Figure 4:**
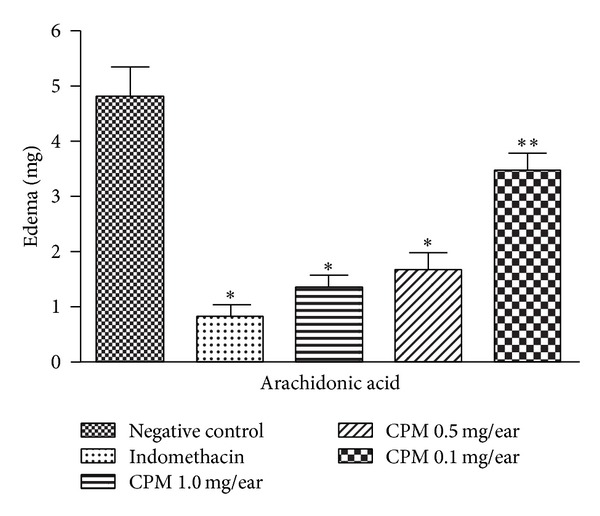
Effect of CPM single application arachidonic acid- (AA-) induced mouse ear edema. Indomethacin was used as positive control. Statistical analysis: one-way ANOVA followed by Newman-Keuls test (*n* = 6–8). ***P < 0.001; **P < 0.01 compared to negative control group.

CPM significantly decreased the ear edema induced by arachidonic acid in a dose-response manner by 72% and 65%, at 1 mg/ear and 0.5 mg/ear, respectively. On the other side, at 0.1 mg/ear, CPM inhibited edema only by 27%. The level of inhibition induced by indomethacin (0.5 mg/kg) was 83% (Figure 4).

#### 3.2.4. Effect of CPM on Capsaicin-Induced Mice Ear Edema

**Figure 5:**
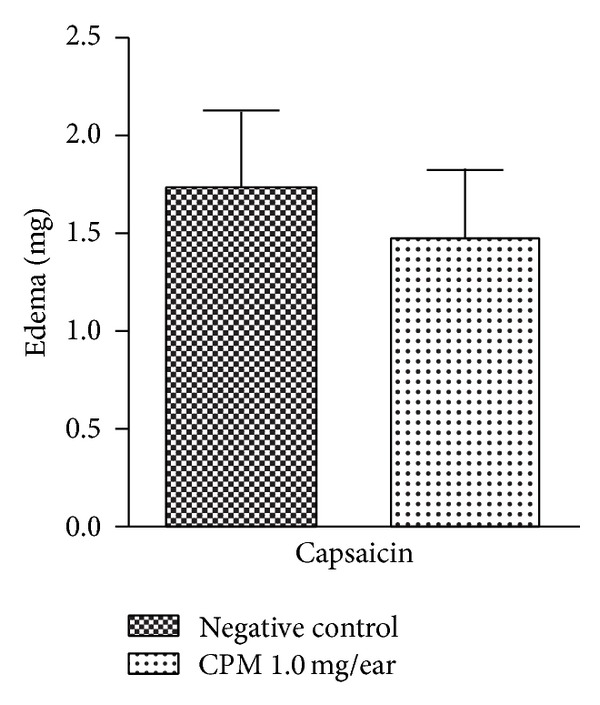
Effect of CPM single application capsaicin-induced mouse ear edema (*n* = 6–8). Statistical analysis: one-way ANOVA followed by Newman-Keuls test.

The topical application of CPM (1 mg/ear) did not present significant reduction on capsaicin-induced ear inflammation (*P* > 0.05) (Figure 5).

#### 3.2.5. Effect of CPM on EPP-Induced Mice Ear Edema

**Figure 6:**
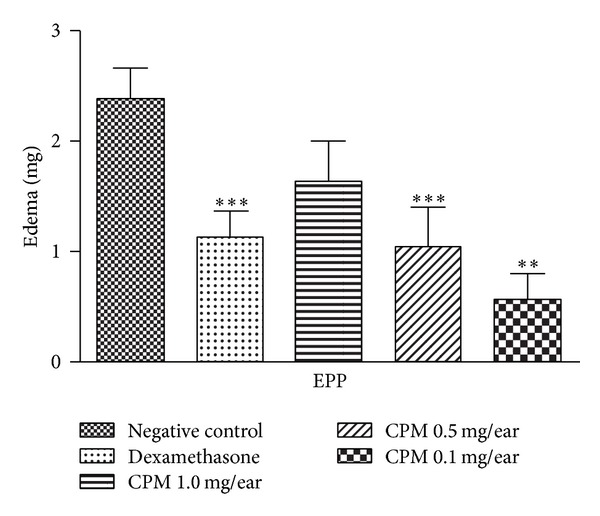
Effect of CPM single application EPP-induced mouse ear edema. Dexamethasone was used as positive control. Statistical analysis: one-way ANOVA followed by Newman-Keuls test (*n* = 6–8). **P < 0.01; *P < 0.05 compared to negative control group.

CPM at 0.5 and 0.1 mg/ear demonstrated a significant reduction of edema when compared to the negative control, 56% and 76%, respectively. It is important to point out that the lowest dose (0.1 mg/ear) was more active than the dexamethasone (0.1 mg/ear) which decreased the edema by 52% (Figure 6).

#### 3.2.6. Effect of CPM on Phenol-Induced Mice Ear Edema

**Figure 7:**
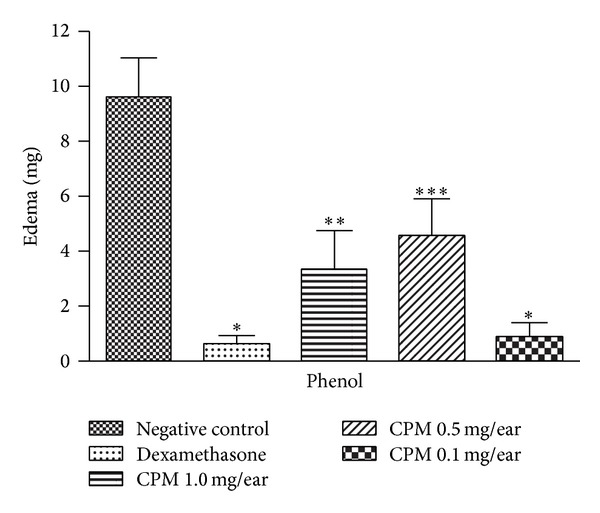
Effect of CPM single application phenol-induced mouse ear edema. Dexamethasone was used as positive control. Statistical analysis: one-way ANOVA followed by Newman-Keuls test (*n* = 6–8). *P < 0.05, **P < 0.01, and ***P < 0.001 compared to negative control group.

There was an intense formation of edema in the ear of the control group 1 h after topical application of phenol. CPM at the lowest dose of 0.1 mg/ear significantly reduced the phenol-induced edema (91%) when compared to dexamethasone (0.1 mg/ear) (97%). Decrease of edema was also observed at 1 mg/ear and 0.5 mg/ear by 65% and 52%, respectively (Figure 7).

#### 3.2.7. Histopathology

**Figure 8:**
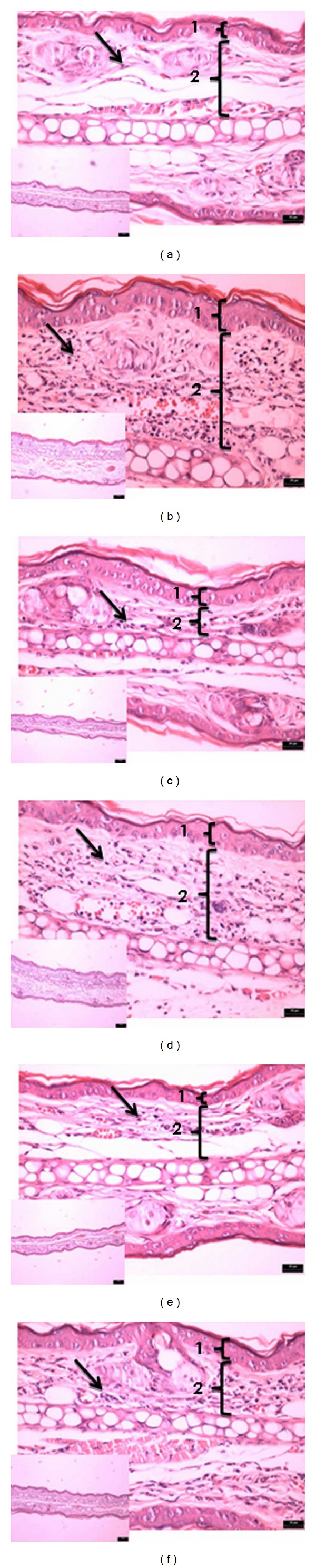
Photomicrograph of transverse sections of mice ears sensitized with topical application of Croton oil 5% (v/v) in acetone (b–f) or vehicle acetone (a, noninflamed), stained with hematoxylin-eosin and examined under light microscopy (magnification: 10x, 40x). Treatments: acetone (b), dexamethasone 0.1 mg/ear (c), EMCP 1.0 mg/ear (d) 0.5 mg/ear (e), and 0.1 mg/ear (f). The numbers 1 and 2 indicate epidermis and dermis, respectively. Arrows indicate leukocyte infiltrate in the dermis. The shown sections are representative of four animals per group.

Histological sections of the ears submitted to a single Croton oil application revealed a significant increase in the dermis thickness, which was accompanied by vascular congestion/vasodilation and infiltration of inflammatory cells (Figure 8(b)). The ears treated with dexamethasone showed a reduction in infiltration of inflammatory cells and lower dermis thickness (Figure 8(c)) when compared to the ear treated with acetone (Figure 8(a)). Histological sections of the ears treated with CPM 0.1 and 0.5 (Figures 8(e) and 8(f), resp.) presented significant reduction of edema and vasodilation and also moderate reduction in infiltration of inflammatory cells comparable to dexamethasone (Figure 8(c)) while CPM at 1 mg/ear (Figure 8(d)) showed slight improvement when compared to control inflamed ear (Figure 8(b)).

**Figure 9:**
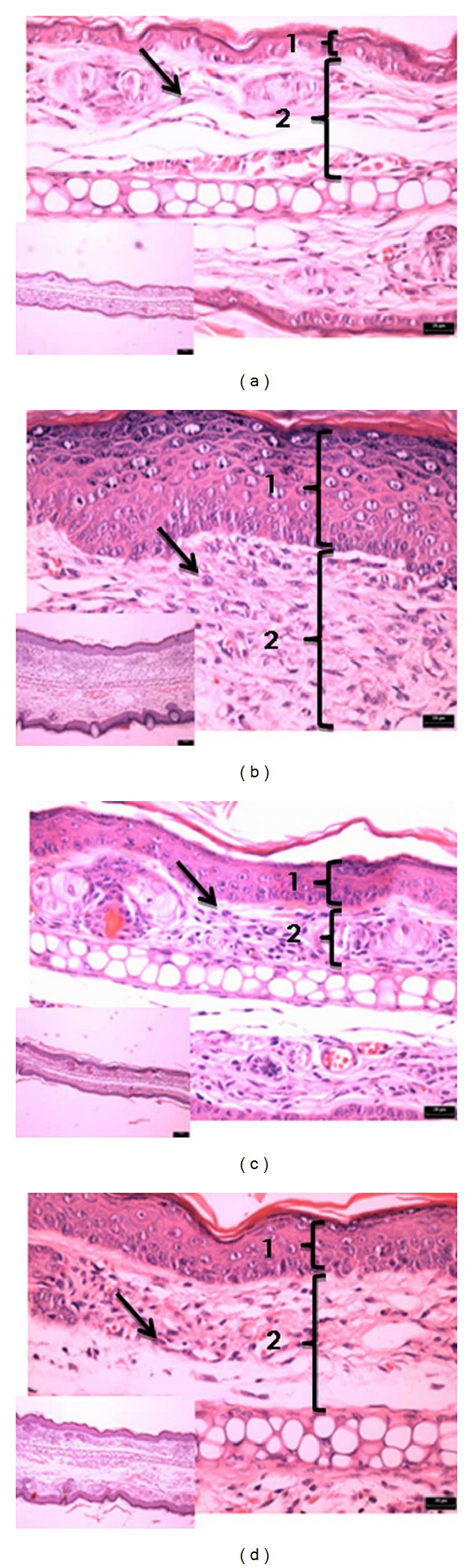
Photomicrograph of transverse sections of mice ears sensitized with multiple topical application of Croton oil 5% (v/v) in acetone (b–d) or vehicle acetone (a, noninflamed), stained with hematoxylin-eosin and examined under light microscopy (magnification: 10x, 40x). Treatments: acetone (b), dexamethasone 0.1 mg/ear (c), and EMCP 0.5 mg/ear (d). The numbers 1 and 2 indicate epidermis and dermis, respectively. Arrows indicate leukocyte infiltrate in the dermis. The shown sections are representative of four animals per group.

Multiple applications of croton oil induced an intense increase in the ear edema, leucocyte infiltration into the dermis with the presence of epidermal alteration characterized by epithelial hyperplasia (acanthosis) associated with hypergranulosis and compact hyperkeratosis (Figure 9(b)).

The positive control dexamethasone (0.1 mg/ear) reduced the inflammatory parameters, such as edema formation and leucocyte infiltration, and thereby markedly reduced the hyperproliferation of keratinocytes caused by repeated croton oil application (Figure 9(c)). In this model, CPM was evaluated only at the dose of 0.5 mg/ear and showed discrete dermal anti-inflammatory activity: however the epidermis showed a decrease in dermal hyperplasia (Figure 9(d)) whose thickness was similar to the positive control group (Figure 9(c)). It is important to point out the occurrence of focal areas of ulceration covered by fibrinonecrotic material with underlying acute inflammatory process.

### 3.3. *In Vitro* Antioxidant Activities of CPM

The* in vitro* antioxidant activity of CPM was examined by employing DPPH free radical scavenging, reducing power, *β*-carotene/linoleic acid bleaching, and thiobarbituric acid reactive substances (TBARS) assays. CPM exhibited strong antioxidant activities in all models used. Results are shown in Table 1.

## 4. Discussion

The mechanism of inflammation injury is attributed, in part, to release of reactive oxygen species from activated neutrophils and macrophages. This over production leads to tissue injury by damaging macromolecules and lipid peroxidation of membranes [24, 25]. Therefore, in this study, the topical anti-inflammatory effects of CPM on acute and chronic cutaneous inflammation models in mice and its* in vitro* antioxidant activity were investigated.

The applications of mouse models of ear edema induced by different irritant agents (croton oil, capsaicin, AA, phenol, and EPP) have long been accepted as useful pharmacological tools for the investigation of new anti-inflammatory drugs, allowing the identification of potential drugs, including substances of plant origin and plant extracts, to treat inflammatory skin disorders and to propose their possible mechanism of action [19]. The use of those irritant agents is important as they induce different inflammation pathways which may indicate distinct mechanisms of action.

Croton oil single application provides data regarding the antiedematous activity in an acute inflammatory process. It contains 12-*o*-tetracanoilphorbol-13-acetate (TPA) whose topical application triggered local inflammation with edema formation, polymorphonuclear leucocytes infiltration, and epidermal hyperproliferation (in the case of croton oil multiple application) as consequence of the production of inflammatory mediators, such as prostaglandin E_2_, leukotrienes, histamine, serotonin, and IL-1 [26]. Corticoid-like agents, phospholipase A_2_, and cyclooxygenase inhibitors as well as 5-lipoxigenase inhibitors and LTB_4_ antagonists are highly effective against the inflammation caused by TPA [19, 26]. CPM at all doses tested was effective in croton oil single application-induced mouse ear edema (Figure 2). Due to the best effect observed for CPM 0.5 mg/ear, this dose was chosen for the croton oil multiple application model which is used to evaluate the antiedematous activity in an established (chronic) inflammatory process [14]. In fact, repeated croton oil application is associated with an increase in ear weight, intense neutrophilic infiltration, macrophages and T cells (CD_4+_ and CD_8+_) migration, and hyperproliferative epidermis (acanthosis). Corticosteroids and 5-LOX inhibitors reduce the edematous effect in this model, while COX inhibitors and antihistamines show little or no effect [27]. Therefore, this model can be used to investigate the involvement of drugs related to release of leukotrienes [14]. In this study, it was observed that while dexamethasone inhibited the edema induced by croton oil multiple application, CPM presented no effect (Figure 3).

The histopathological analysis corroborated the acute response of CPM observed in the croton oil simple application model (Figures 8(a)–8(f)). On the other side, histopathology of groups submitted to the chronic inflammation model and treated with dexamethasone and CPM at 0.5 mg/ear showed moderate anti-inflammatory effectiveness with considerable reduction in the epidermal hyperplasia in areas without ulceration, contrasting with severe epidermal hyperplasia in the group of untreated animals (negative control) (Figures 9(a)–9(d)). This difference can be explained by the fact that during the inflammatory process some inflammatory factors (like cytokines) are released or by the cells of the altered tissue either by the inflammatory infiltrate, which act as mediator factors that possibly influence the epidermal growth [28–30]. It means that the quality and quantity of the dermal inflammatory infiltrate and growth factors released can justify the decrease in epidermal thickness observed in the groups treated with dexamethasone and CPM 0.5 mg (Figures 9(a)–9(d)).

The results observed in histopathological analysis suggested an indirect anti-inflammatory action of CPM, even with ear thickness unchanged. The areas of ulceration observed in some animals of all groups in the model of chronic inflammation may be caused by the animals self-scratching or scratches by other animal in the same cage, or even due to repeated application of croton oil. The ulcer of the epidermis triggered a process of tissue repair initiated by the installation of an acute inflammatory process. This may explain the acute inflammation (associated with marked edema, vasodilation, vascular congestion, and leukocyte infiltration) observed in some areas of the ears treated with CPM and also may explain why no decrease in ear thickness was observed, mainly in the group treated with CPM 0.5 mg/ear (Figure 9(d)).

Topical application of AA provokes a rapid and intense inflammatory response in the mouse ear [15, 31]. AA is a precursor of inflammatory eicosanoids such as prostaglandin E_2_ (PGE_2_) and leukotrienes (produced via COX-1, COX-2, and 5-LOX enzymes). COX and LOX inhibitors, like indomethacin, classically cause significant reduction in AA-induced ear edema [19, 32]. AA metabolites are also involved in mast cells degranulation, which is accompanied by histamine release. Consequently, antihistamines are also able to reduce AA-induced edema [15, 31, 33]. CPM significantly decreased the ear edema in this model (Figure 4).

Application of capsaicin, the primary pungent ingredient of red peppers, to the ear of mouse produces neurogenic skin inflammation [16]. When it contacts with epidermis, capsaicin activates the TRPV1 receptor, which elicits a rapid response via the release of neuropeptides (such as calcitonin gene related peptide, substance* P,* and tachykinins) and monoamimes (histamine and serotonin) [16, 34, 35]. It induces many signs of acute inflammation, for example, vasodilation [36] resulting in a flare reaction, increased blood flow [37], and elevated temperature [38]. The topical application of CPM did not present significant reduction on capsaicin-induced ear inflammation, suggesting that CPM did not interfere in substances or receptors involved in capsaicin-activated inflammatory pathways (Figure 5)

EPP induces edema formation due to vasodilation and an increase on vascular permeability. This event is caused by the release of several inflammatory mediators, such as histamine, serotonin, bradykinin, and prostaglandins [39]. CPM markedly inhibited the formation of EPP-ear induced edema.

Topical application of phenol directly affects the skin by inducing inflammation and tissue damage [40–42]. Keratinocyte membranes are ruptured, resulting in release of cytokines such as IL-1*α*, TNF-*α*, and IL-8 by a mechanism independent on PKC activation pathway (as what occurs in inflammations induced by croton oil), which in turn results in release of other inflammatory mediators such as AA metabolites and reactive oxygen species (ROS) [43, 44]. Severe edema, erythema, and necrosis occur as a result of the application ofphenol [45, 46]; CPM significantly reduced the phenol-induced edema in the lowest dose of 0.1 mg/ear (Figure 7) when compared to dexamethasone, the positive control, which also modulates the production of AA metabolites, probably via inhibition of COX and LOX enzymes or via production of anti-inflammatory eicosanoids [47]. This finding can also indicate the potential use of CPM in irritant contact dermatitis.

Plant extracts are endowed with a variety of compounds which may act in distinct pathways. This could, in part, explain the non-dose dependent response found when using different inflammation models. Another possible explanation is that in noncompetitive antagonism, the threshold dose of agonist is not markedly increased, but the maximum response is depressed in the presence of the antagonist [48].

CPMwas found to possess a strong antioxidant and free radical scavenging activity when tested in DPPH, reducing power, *β*-carotene bleaching, and lipid peroxidation assays. It showed different behaviour in the four* in vitro* assays (Table 1), probably due to the different mechanisms involved in the steps of the oxidation process. It has been assumed that ROS also play an important role in the edema formation in some of these models, so the antioxidant activity of CPM may contribute to the anti-inflammatory activity found for this extract.

As the antioxidant activity measured by an individual assay reflects only the chemical reactivity under specific conditions applied in that assay, it is inappropriate and misleading to use a single method to evaluate antioxidant capacity of a sample [49]. Interestingly, CPM was shown to contain phenolic substances as chlorogenic acid, orientin, and isoorientin (Figure 1).

Plants polyphenols are secondary metabolism products and they constitute one of the most numerous and widely distributed groups of natural antioxidants. Those compounds act as reducing agents and antioxidants* in vitro* via several mechanisms including the scavenging of free radicals, chelation of transition metals, and the mediation and inhibition of enzymes [50].

## 5. Conclusions

In conclusion, our study has demonstrated the relevant topical antiedematous effect of CPM in mouse ear edema induced by different agents, which can be the consequence of combined antioxidant and anti-inflammatory effect, including COX inhibition, and indicated its potential application as a herbal medicine to be used on skin inflammatory diseases. The antiproliferative effect on the epithelium cells, observed histopathologically for CPM in the model of chronic inflammation, although secondary, should be further investigated aiming its application in chronic dermatoses associated with acanthosis, such as psoriasis. Moreover, further efforts are still needed for the isolation, characterization, and biological evaluation of the active(s) principle(s) of CPM.

## Figures and Tables

**Table 1:** Antioxidant activity determined by DPPH assay, reducing power, *β*-carotene/acid linoleic, and TBARS methods.

Species and positive controls	DPPH scavenging activity (IC_50_—µg/mL)^a^	Reducing power (EC_50_—µg/mL)^a^	*β*-carotene/acid linoleic system (IC_50_—µg/mL)^a^	TBARS (% inhibition)^a^
CPM	3.1 (±0.25)*	10.85 (±0.65)*	5.1 (±1.31)*	81 (±0.04)
Quercetin	0.98 (±0.20)	(—)	1.19 (±0.62)	(—)
Rutin	2.10 (±0.60)	(—)	(—)	(—)
Ascorbic acid	(—)^b^	1.80 (±0.48)	(—)	(—)
BHT	(—)	(—)	(—)	88 (±0.01)

^a^Values expressed as mean ± S.D. of triplicates testes.

^b^Not determined.

*Statistically different (*P* < 0.005) from positive control group (quercetin and rutin in DPPH test, ascorbic acid in reducing power, and quercetin in *β*-carotene/acid linoleic system) (ANOVA followed by test *t*-Student).
